# *SLC2A10 *genetic polymorphism predicts development of peripheral arterial disease in patients with type 2 diabetes. *SLC2A10 *and PAD in type 2 diabetes

**DOI:** 10.1186/1471-2350-11-126

**Published:** 2010-08-25

**Authors:** Yi-Der Jiang, Yi-Cheng Chang, Yen-Feng Chiu, Tien-Jyun Chang, Hung-Yuan Li, Wen-Hsing Lin, Hsiang-Yu Yuan, Yuan-Tsong Chen, Lee-Ming Chuang

**Affiliations:** 1 Department of Internal Medicine, National Taiwan University Hospital, Taipei, Taiwan; 2 Genomics Research Center, Academia Sinica, Taipei, Taiwan; 3 Division of Biostatistics and Bioinformatics, National Health Research Institutes, Zhunan, Taiwan; 4 Division of Biotechnology and Pharmaceutical Research, National Health Research Institutes, Zhunan, Taiwan; 5 Institute of Biomedical Sciences, Academia Sinica, Taipei, Taiwan; 6 Graduate Institute of Clinical Medicine, National Taiwan University College of Medicine, Taipei, Taiwan

## Abstract

**Background:**

Recent data indicate that loss-of-function mutation in the gene encoding the facilitative glucose transporter GLUT10 (*SLC2A10*) causes arterial tortuosity syndrome via upregulation of the TGF-β pathway in the arterial wall, a mechanism possibly causing vascular changes in diabetes.

**Methods:**

We genotyped 10 single nucleotide polymorphisms and one microsatellite spanning 34 kb across the *SLC2A10 *gene in a prospective cohort of 372 diabetic patients. Their association with the development of peripheral arterial disease (PAD) in type 2 diabetic patients was analyzed.

**Results:**

At baseline, several common SNPs of *SLC2A10 *gene were associated with PAD in type 2 diabetic patients. A common haplotype was associated with higher risk of PAD in type 2 diabetic patients (haplotype frequency: 6.3%, *P *= 0.03; odds ratio [OR]: 14.5; 95% confidence interval [CI]: 1.3- 160.7) at baseline. Over an average follow-up period of 5.7 years, carriers with the risk-conferring haplotype were more likely to develop PAD (*P *= 0.007; hazard ratio: 6.78; 95% CI: 1.66- 27.6) than were non-carriers. These associations remained significant after adjustment for other risk factors of PAD.

**Conclusion:**

Our data demonstrate that genetic polymorphism of the *SLC2A10 *gene is an independent risk factor for PAD in type 2 diabetes.

## Background

Peripheral arterial disease (PAD), defined as lower extremity arterial atherosclerosis, is one of most common diseases of the arteries and is a major complication of type 2 diabetes [1]. Conventional cardiovascular risk factors such as aging, smoking, hyperglycemia, hypertension and dyslipidemia have been shown to be associated with PAD [1]. However, the increased risk for atherosclerotic diseases in diabetic patients can be only partially explained by the conventional risk factors [2]. In fact, a high heritability for ankle-brachial blood pressure index (ABI), an index of PAD, has been obtained in Twin studies in Caucasians [3], indicating that additional genetic factors might be involved in the pathogenesis of PAD. In this respect, the search for genetic causes of PAD remains limited [4].

Recently, a genetic form of arterial tortuosity syndrome (ATS; OMIM 208050) was reported to be caused by loss-of-function mutations in the *SLC2A10 *gene encoding the facilitative glucose transporter GLUT10. Affected individuals are characterized by tortuosity of the large and medium-sized arteries, and often resulting in premature death at a young age [5-8]. The features of vascular changes observed in ATS were similar to those described for Loeys-Dietz syndrome (LDS; OMIM 609192), characterized by upregulation of transforming growth factor (TGF)-β signaling [5]. TGF-β mediates the downstream the signaling of hyperglycemia-activated protein kinase C and increases vascular extracellular matrix deposition in vessels exposed to hyperglycemia [9]. Increased TGF-β signaling also correlates with the microangiopathic changes and fibrosis seen in diabetic retinopathy, nephropathy, and peripheral arterial disease [9-11]. These data indicate that the *SLC2A10 *gene is a candidate gene of vascular complications in subjects with type 2 diabetes.

To explore the association of *SLC2A10 *genetic polymorphism with PAD in type 2 diabetic patients, we recruited a total of 372 diabetic patients from a Taiwanese population. These patients were followed for the development of PAD over an average period of 5.7 years. The associations of *SLC2A10 *genetic variants with baseline and incident PAD during follow-up were analyzed.

## Methods

### Subjects and Phenotype Measurements

This study recruited 372 patients with type 2 diabetes diagnosed with the WHO criteria of 1998 [12] from the metabolic clinic of the National Taiwan University Hospital, Taipei, Taiwan. All subjects are Han Chinese by self-report. Body weight and height were measured to calculate body mass index (BMI). Seated blood pressure was measured after at least 5 min of resting. Demographic data and past medical history including cardiovascular, cerebrovascular and peripheral arterial diseases were documented. PAD was defined as an ankle-brachial index (ABI) < 0.9 in any of the two limbs, using a handheld Doppler ultrasound (Medacord PVL, Medasonics, Fremont, CA) over brachial and dorsalis pedis or posterior tibial pulses according to the American College of Cardiology/American Heart Association guideline [13]. The concentrations of plasma glucose, total cholesterol, and triglyceride were measured in fasting samples by an autoanalyzer (Hitachi 7250 special, Tokyo, Japan). HbA1c was measured with high-performance liquid chromatography (CLC385, Primus Corporation, Kansas City, MO). All subjects were unrelated and were given written informed consent. The study protocol was approved by the Institutional Review Board of the National Taiwan University Hospital. All participants and investigators were blind to the genotyping information.

### SNP Selection and Genotyping

Genomic DNA was isolated using the PUREGENE DNA purification system (Gentra Systems, Minneapolis, MN). A total of 11 polymorphic markers, including 10 SNPs (rs2425895, rs2143044, rs3092412, rs2235491, rs2425904, rs2425911, rs3091904, rs1059217, rs6066059, rs2179357) and one (TGTGTGTGT)*n *microsatellite, were selected using the SNPper software http://snpper.chip.org and were genotyped as previously described [14]. Samples with failed genotyping were analyzed with direct sequencing and therefore the genotype success rate was 100%. The genotyping concordance rate based on 92 replicates was 98.9%.

### Statistical Analysis

Hardy-Weinberg equilibrium tests were carried out before conducting marker-trait association analyses. Inter-marker linkage dis-equilibrium (LD) was estimated by measuring pairwise D' and *r*^2 ^and were displayed using the Haploview software [15]. The SAS/Genetics package was used for the single-locus association analysis and allele and genotype *p*-values were calculated from Chi-square or Fisher exact tests. Odds ratios (OR) and 95% confidence intervals (CI) were estimated with multivariate logistic regression. For haplotype analyses, we used the score test developed by Schaid *et al*. and implemented in the Haplo. Stats package http://mayoresearch.mayo.edu/schaid_lab/software.cfm[16]. This method allows the adjustment for possible confounding variables. Haplotypes with frequencies less than 0.01 were grouped into rare haplogroups in the regression analyses. Nominal two-sided *P*-values were calculated with simulation for 1,000 times. Cox proportional hazard models were used to evaluate the risk of developing PAD by carrying individual haplotypes. The hazard ratios (HR) comparing the risks of developing PAD between individuals with and without each specific haplotype were computed. Potential confounders including age, sex, duration of diabetes, BMI, triglyceride, total cholesterol, HbA1c, systolic blood pressure and current smoking status were adjusted in these analyses. The Kaplan-Meier method was employed to estimate the survival curves for individuals with and without a specific haplotype. The log-rank test was used to assess the significance level of the difference between two curves. These analyses were performed using LIFETEST procedure in the SAS statistical package.

## Results

The baseline characteristics of study participants are summarized in Table 1. At baseline, 27 (7.26%) of 372 type 2 diabetic subjects had PAD. There were significant differences between patients with or without PAD in age, duration of diabetes, and systolic blood pressure. No significant difference was found for other variables between the two groups.

**Table 1:** Characteristics of type 2 diabetic patients according to absence (-) or presence (+) of PAD at baseline.

	PAD (-) N = 345	PAD (+) N = 27	*P*-value
Age (year)	60.2 ± 11.5	71.4 ± 8.11	**< 0.0001**
Duration of diabetes (year)	10.7 ± 7.63	16.4 ± 11.2	**0.01**
Sex (male: female)	177:168	17:10	0.24
BMI (kg/m^2^)	24.9 ± 3.32	24.7 ± 3.60	0.86
Triglyceride (mmol/l)	1.74 ± 0.93	1.74 ± 0.93	0.99
Total cholesterol (mmol/l)	5.15 ± 1.01	5.32 ± 0.88	0.40
Hemoglobin A1c (%)	7.67 ± 1.39	7.95 ± 1.13	0.30
Systolic blood pressure (mmHg)	134.3 ± 16.5	145.0 ± 16.5	**0.003**
Smoker: non-smoker	269:76	21:6	0.98

**Figure 1:**
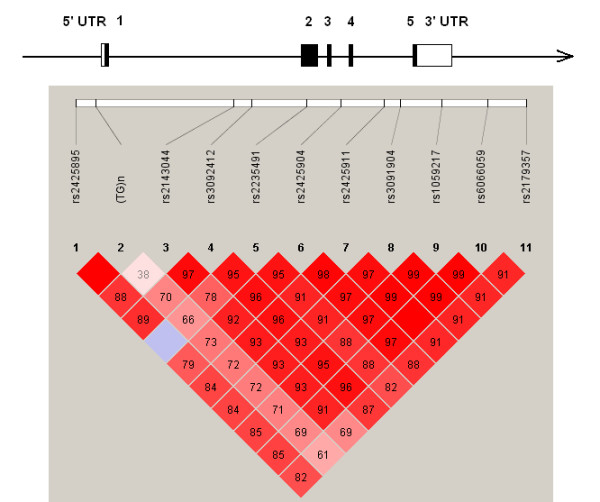
**Graphical representation of SNPs in relation to the exon-intron structure (upper part) and Haploview LD graph of *SCL2A10 *gene (lower part)**. The exon regions are shown with filled rectangles and numbered in order. Pairwise LD coefficients D' × 100 was shown in each cell (D' values of 1.0 were not shown). Standard color scheme of Haploview was applied for LD color display (LOD score > 2 and D' = 1 in bright red; LOD score > 2 and D' <1 in blue; LOD score <2 and D' = 1 in shade of pink; LOD score <2 and D' <1 in white).

Eleven polymorphic markers including 10 SNPs and one microsatellite spanning 34 kb across the *SLC2A10 *gene were genotyped. All markers were in Hardy-Weinberg equilibrium (data not shown). Graphical representations of these markers in relation to the exon-intron structure and the LD structure are shown in Figure 1. The genomic position, gene region, and nucleic acid compositions of the markers are summarized in Table 2.

**Table 2:** *SLC2A10 *sequence variants and association with PAD in type 2 diabetic patients at baseline.

#	ID	Position (kb)	Function	Major/ minor allele	Minor allele frequency PAD(-) PAD(+)		Allelic *p*-value (adjusted*)	Allele OR* (95% CI)	Genotype distribution** PAD (-) PAD (+)		Genotypic *p*-value (adjusted*)
1	rs2425895	-1.789	5' upstream	G/A	0.067	0.056	0.75 (0.83)	0.87 (0.24-3.13)	301:42:2	24:3:0	1.00 (0.82)
2	(TGTGTGTGT)_n_	-0.208	5' upstream	3/2	0.24	0.28	0.54 (0.38)	1.37 (0.68-2.80)	198:128:18	14:11:2	0.72 (0.39)
3	rs2143044	10.341	Intron 1	C/T	0.31	0.44	**0.03 (0.03)**	2.00 (1.04-3.85)	167:141:35	7:16:4	0.05 (0.04)
4	rs3092412	11.720	Intron 1	A/T	0.39	0.56	**0.01 (0.005)**	2.51 (1.31-4.81)	130:164:51	4:16:7	**0.03 (0.006)**
5	rs2235491	15.915	Exon 2 Ala206Thr	G/A	0.074	0.13	0.14 (0.036)	3.03 (1.08-8.55)	296:47:2	21:5:1	0.11 (0.05)
6	rs2425904	18.500	Intron 3	T/C	0.37	0.56	**0.007 (0.004)**	2.58 (1.34-4.98)	133:167:45	3:18:6	**0.006 (0.004)**
7	rs2425911	21.843	Intron 4	G/C	0.38	0.56	**0.01 (0.005)**	2.54 (1.32-4.87)	132:164:49	3:8:6	**0.009 (0.005)**
8	rs3091904	23.079	Intron 4	C/T	0.38	0.57	**0.004 (0.002)**	2.83 (1.46-5.46)	136:158:51	3:17:7	**0.005 (0.002)**
9	rs1059217	26.273	3' UTR	C/T	0.37	0.56	**0.007 (0.004)**	2.58 (1.35-4.95)	185:160:0	9:18:0	**0.005 (0.004)**
10	rs6066059	29.778	3' downstream	C/T	0.37	0.56	**0.005 (0.004)**	2.61 (1.36-5.00)	138:161:46	3:18:6	**0.004 (0.004)**
11	rs2179357	32.708	3' downstream	C/T	0.33	0.57	**2.6 × 10^-4 ^(< 0.0001)**	3.87 (1.97-7.58)	160:143:42	4:15:8	**0.001 (< 0.0001)**

*P-values and odds ratios were adjusted for covariates including age, sex, duration of diabetes, BMI, triglyceride, total cholesterol, HbA1c, systolic blood pressure and current smoking status.

**Genotype distribution are shown as number (AA: AB: BB); A represents the major allele and B represents the minor allele.

PAD, peripheral artery disease; OR, odds ratio; CI, confidence interval

We first compared the allele frequencies between type 2 diabetic subjects with and without PAD at baseline. Eight SNPs showed nominally significant associations with PAD in type 2 diabetes (Table 2). Among them, the T allele at rs2179357 showed the strongest association with PAD in type 2 diabetic patients (*P *= 2.6 × 10^-4^; OR: 3.87; 95% CI: 1.97- 7.58) (Table 2), which remained significant after applying the Bonferroni correction for multiple testing. The genotypic odds ratio was 7.62 (95%CI: 1.91-35.8, *P *= 0.0003) for the homozygous genotype (TT) and was 4.20 (95% CI: 1.91-7.58, *P *= 0.007) for the heterozygous genotype (TC). Combining all 11 markers, we identified a common haplotype (H4) conferring a strong risk of PAD in type 2 diabetic patients (*P *= 0.03, OR: 14.5; 95% CI: 1.3-160) (Table 3). At baseline, 6 of the 27 subjects with PAD were estimated to be H4 haplotype carriers, whereas only 39 of the 345 subjects without PAD carried the H4 haplotype. These associations remained significant after adjustment for possible confounding risk factors including age, sex, systolic blood pressures, hemoglobin A1C (HbA1C) levels, durations of diabetes, BMI, current smoking status, serum triglyceride, and total cholesterol levels (Table 4), indicating that the *SLC2A10 *genetic polymorphism is an independent risk factor of PAD in type 2 diabetes.

**Table 3:** *SLC2A10 *haplotype analysis for association with baseline, incident, and all PAD in type 2 diabetes.

	Haplotype Sequence	Frequency	Baseline PAD association		Incident PAD association	
			P^* ^	OR (95% CI)*	P^* ^	HR (95%CI)*
H1	A3TAGCCTTTT	0.051	0.87	1.28(0.07-22.8)	0.89	1.17(0.13-10.4)
H2	G3CTGTGCCCC	0.499	**0.01**	0.20(0.05- 0.75)	0.80	1.20(0.30- 4.72)
H3	G2TAGCCTTTT	0.118	0.08	4.20(0.82- 21.6)	0.29	0.42(0.09- 2.06)
H4	G3TAGCCTTTT	0.063	**0.03**	14.5(1.3-160)	**0.007**	6.78(1.66- 27.6)
H5	G2CAACCTTTT	0.041	0.97	1.07(0.04- 29.5)	0.99	1.8 × 10^-7 ^(0-∞)
H6	G2CTGTGCCCC	0.026	0.42	5.4 × 10^-4^(0- 40159)	0.66	0.60(0.06-5.99)
H7	G3TAGCCTTTC	0.023	0.48	1.7 × 10^-6 ^(0-∞)	0.87	1.22(0.10- 14.5)
H8	G3CTGTGCCCT	0.015	0.40	7.67(0.07- 894)	0.34	3.01(0.31- 29.0)
Rare haplotypes		0.15	0.26	1.36(0.80-2.31)	0.99	4.8 × 10^-6 ^(0-∞)

^* ^*P*-values, odds ratios, and hazard ratios were adjusted for covariates including age, sex, duration of diabetes, body mass index, triglyceride, total cholesterol, hemoglobin A1C, systolic blood pressure and current smoking status.

PAD, peripheral artery disease; OR, odds ratio; HR, hazard ratio, CI, confidence interval

**Table 4:** Multivariate regression analyses of the *SLC2A10 *haplotype H4 with PAD in type 2 diabetes.

Risk factors	Effect estimate	OR (95% CI)	*P*-values
Age (per year)	0.0812	1.09(1.04,1.13)	**3 × 10^-4^**
Male	-0.0456	0.91(0.38,2.19)	0.83
Hemoglobin A1C (per 1%increase)	0.1046	1.11(0.91,1.36)	0.31
Duration of diabetes (per year)	0.0467	1.05(1,1.1)	**0.04**
Triglyceride (per increase of 1mM)	0.2118	1.24(0.79,1.92)	0.34
Total cholesterol (per increase of 1mM)	-0.1952	0.82(0.54,1.26)	0.36
Systolic blood pressure (per 1 mmHg increase)	0.0463	1.05(1.03,1.07)	**1.9 × 10^-5^**
Current smoker	-0.2913	0.56(0.22,1.44)	0.22
Body mass index (per kg/m^2 ^increase)	-0.0564	0.95(0.83,1.08)	0.39
H4 haplotype	3.4171	30.47(4.47-208.5)	**5 × 10^-4^**

PAD, peripheral artery disease; OR, odds ratio; CI, confidence interval

**Figure 2:**
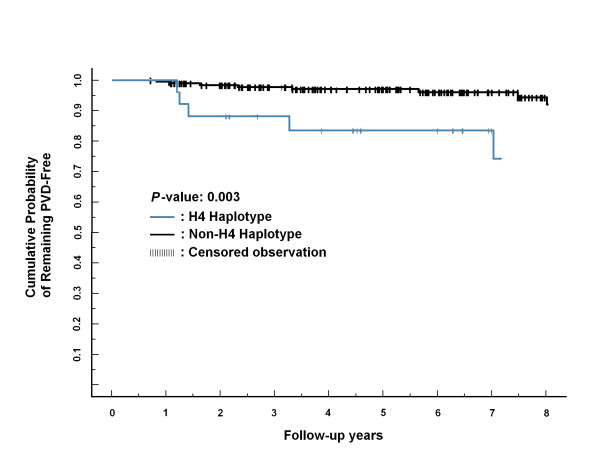
**Cumulative probability of PAD in type 2 diabetic patients with and without haplotype H4**.

We next examined whether the risk haplotype affected the incidence of PAD in type 2 diabetic subjects. Over an average follow-up period of 5.7 years, 13 (3.76%) of the 345 diabetic patients without PAD at the baseline developed PAD. Carriers of risk-conferring haplotype H4 were more likely to develop PAD than were other haplotypes during follow-up (*P *= 0.007; HR: 6.78; 95% CI: 1.66-27.6) (Table 3). During follow-up, 5 of the 13 incident PAD cases were estimated to be H4 carriers, whereas H4 haplotype was only present in 34 of the 332 subjects who did not develop PAD. The Kaplan-Meier curves representing the cumulative incidence of PAD for individuals with and without carrying the haplotype H4 is depicted in Figure 2.

To further search for causative variants of *SLC2A10*, we performed the direct DNA sequencing of all exons and flanking intronic sequences of the *SLC2A10 *gene in all patients with PAD. No pathogenic mutations other than the aforementioned SNPs were found (data not shown).

## Discussion

This study showed that common *SLC2A10 *genetic variants were associated with the development of PAD in type 2 diabetic patients independent of all known risk factors for PAD including age, sex, smoking, lipids, and blood pressures. The association was observed at baseline and was further replicated during follow-up study. These data suggest the *SLC2A10 *gene plays a significant role in the pathogenesis of PAD in diabetic patients.

The *SLC2A10 *gene encodes GLUT10, a facilitative glucose transporter. The facilitated glucose transporter (GLUT) mediated the uptake of several monosaccharides including glucose, fructose, mannose, galactose, and glucosamine [17]. The physiological function of GLUT10 was unclear until recently, when a genetic form of arterial tortuosity syndrome was identified by a loss-of-function mutation in the *SLC2A10 *gene [5-8], with a pathology characterized by upregulation of TGF-β signaling in the vascular walls. TGF-β signaling is one of the final common pathways linking hyperglycemia and vascular complications in individuals with diabetes mellitus [9]. In addition, impaired uptake of monosaccharides, a function of GLUT10, may hinder glycosylation, which is an important step for the production of functional glycoproteins and proteoglycans [5]. Glycoproteins and proteoglycans are essential structural components of the arterial wall and connective tissue. In support of this notion, we recently demonstrated that mutation in the *SLC2A10 *gene in mice causes irregular shape of large and medium-sized arteries, characterized by markedly increased elastic fibers and intimal endothelial hypertrophy [18]. These data further support the role of *SLC2A10 *gene in the pathogenesis of diabetic vascular complications.

The SNPs with significant associations with PAD in our study are located in non-coding regions within a single LD block spanning from intron 1 to the 3' downstream of *SLC2A10 *gene. One non-synonymous SNP rs2235491 (Ala260Thr, exon 2) was in strong LD with rs2179357 (D' = 0.82) but did not show significant association with PAD, which may be attributed to its low frequency in the Taiwanese population. Previous study has provided evidence that the rs2235491 Ala/Ala carriers exhibit higher fasting plasma insulin level and higher area-under-curve of insulin levels after glucose loading [19]. The rs2235491 Ala allele might contribute to the vascular complication in type 2 diabetes via associated hyperinsulinemia. A larger sample size would be required to clarify whether this putatively functional variation is the causal variant. In our study, the association is strongest for the SNPs that are located near the 3' UTR, downstream of the *SLC2A10 *gene. However, bioinformatics analysis of the sequences surrounding this region failed to reveal any conserved regulatory motif among different species to support the functional consequences [20].

Our results demonstrate that variation of the *SLC2A10 *gene not only results in a rare vascular syndrome but also is associated with susceptibility to common vascular complications in type 2 diabetes. It is interesting to note that prevalence of PAD and amputation rate in type 2 diabetes is lower in Chinese populations as compared to Caucasians and Indians [21-24]. Using data from the HapMap database, the risk at the rs2179357 T allele, which is in strong LD with other risk alleles in our study, is present in 81% of Caucasians but only in 44% of Han Chinese [24]. Whether this difference explains the lower incidence of PAD in type 2 diabetic Asians remains to be further studied.

There are certain limitations of this study. First, although a total of 1,967 person-years of follow-up were accumulated in this study, the sample size is relatively small and the incident cases were limited. Therefore, the association may be seriously influenced by random fluctuation. We calculated the power of this study to detect variants with various frequencies and odds ratio. For 345 controls and 27 cases, we will have a statistical power of 0.8 to detect an odds ratio of 3.0, 3.6, 4.6 and 10.0 for a target allele or haplotype frequency of 0.2, 0.1, 0.05 and 0.01, respectively, assuming a type I error rate of 0.05 (Additional File 1: Supplemental Figure S1). Second, the result has not been replicated in an independent study. Although the association in baseline was replicated in longitudinal follow-up, it cannot be viewed as a true independent replication. Thirdly, population stratification may arise when samples are drawn from structure populations. However, our study participants were all Han Chinese by self-report. Previous research has demonstrated that genotype distribution in Han Chinese in Taiwan is highly homogenous [25]. The high homogeneity mitigates the possibility of spurious association. Lastly, the drop-out rate in this study is substantial (31.11% in H4 haplotype carriers and 31.11% in non-H4 haplotype carriers). Although there is no difference in drop-out rate or drop-out reasons between two groups (data not shown), whether the high drop-out rate introduces bias to this study is not known.

## Conclusions

We identified common *SLC2A10 *genetic variants conferring strong and independent risk on development of PAD in type 2 diabetic patients. The present report indicates an important role of the GLUT10 transporter in PAD development in diabetic patients. Whether or not the *SLC2A10 *genetic polymorphism contributes to other micro- or macrovascular complications merits further investigation.

## Competing interests

The authors declare that they have no competing interests.

## Authors' contributions

YDJ, YCC, and YFC participated in the design of the study, analysis of results, and manuscript writing. TJC and HYL provided the sample and clinical data of study participants. WHL finished the genotyping work. HYY and YTC participated in the interpretation of study. LMC participated in the design of the study, interpretation of results, and manuscript revision. All authors read and approved the final manuscript.

## Pre-publication history

The pre-publication history for this paper can be accessed here:

http://www.biomedcentral.com/1471-2350/11/126/prepub

## Supplementary Material

Additional file 1 **Supplemental Figure S1**. The statistical power of current study to detect a risk allele or haplotype with different odds ratios and frequencies with type I error rate of 0.05. Click here for file

## References

[B1] Adler AI Stevens RJ Neil A Stratton IM Boulton AJ Holman RR UKPDS 59: hyperglycemia and other potentially modifiable risk factors for peripheral vascular disease in type 2 diabetes Diabetes Care 2002 25 894 899 10.2337/diacare.25.5.894 11978687

[B2] Keen H Clark C Laakso M Reducing the burden of diabetes: managing cardiovascular disease Diabetes Metab Res Rev 1999 15 186 196 10.1002/(SICI)1520-7560(199905/06)15:3<186::AID-DMRR30>3.0.CO;2-5 10441041

[B3] Carmelli D Fabsitz RR Swan GE Reed T Miller B Wolf PA Contribution of genetic and environmental influences to ankle-brachial blood pressure index in the NHLBI Twin Study. National Heart, Lung, and Blood Institute Am J Epidemiol 2000 151 452 458 10707913 10.1093/oxfordjournals.aje.a010230

[B4] Gudmundsson G Matthiasson SE Arason H Johannsson H Runarsson F Bjarnason H Localization of a Gene for Peripheral Arterial Occlusive Disease to Chromosome 1p31 Am J Hum Genet 2002 70 586 592 10.1086/339251 11833003 PMC384938

[B5] Coucke PJ Willaert A Wessels MW Callewaert B Zoppi N De Backer J Mutations in the facilitative glucose transporter GLUT10 alter angiogenesis and cause arterial tortuosity syndrome Nat Genet 2006 38 452 457 10.1038/ng1764 16550171

[B6] Drera B Guala A Zoppi N Gardella R Franceschini P Barlati S Two novel SLC2A10/GLUT10 mutations in a patient with arterial tortuosity syndrome Am J Med Genet A 2007 143 216 218 17163528 10.1002/ajmg.a.31514

[B7] Zaidi SH Meyer S Peltekova VD Lindinger A Teebi AS Faiyaz-Ul-Haque M A novel non-sense mutation in the SLC2A10 gene of an arterial tortuosity syndrome patient of Kurdish origin Eur J Pediatr 2008 in press 18818946 10.1007/s00431-008-0839-2

[B8] Callewaert BL Willaert A Kerstjens-Frederikse WS De Backer J Devriendt K Albrecht B Arterial tortuosity syndrome: clinical and molecular findings in 12 newly identified families Hum Mutat 2008 29 150 158 10.1002/humu.20623 17935213

[B9] Brownlee M The Pathobiology of Diabetic Complications. A Unifying Mechanism Diabetes 2005 54 1615 1625 10.2337/diabetes.54.6.1615 15919781

[B10] Ziyadeh FN Mediators of Diabetic Renal Disease: The case for TGF-β as the major mediator J Am Soc Nephrol 2004 15 S55 S57 10.1097/01.ASN.0000093460.24823.5B 14684674

[B11] Chiarelli F Santilli F Mohn A Role of growth factors in the development of diabetic complications Hormone Research 2000 53 53 67 10.1159/000023515 10971090

[B12] Alberti KG Zimmet PZ Definition, diagnosis and classification of diabetes mellitus and its complications. Part 1:diagnosis and classification of diabetes mellitus provisional report of a WHO consultation Diabet Med 1998 15 539 553 10.1002/(SICI)1096-9136(199807)15:7<539::AID-DIA668>3.0.CO;2-S 9686693

[B13] Hirsch AT Haskal ZJ Hertzer NR Bakal CW Creager MA Halperin JL ACC/AHA 2005 Guidelines for the Management of Patients With Peripheral Arterial Disease (Lower Extremity, Renal, Mesenteric, and Abdominal Aortic): Executive Summary A Collaborative Report From the American Association for Vascular Surgery/Society for Vascular Surgery, Society for Cardiovascular Angiography and Interventions, Society for Vascular Medicine and Biology, Society of Interventional Radiology, and the ACC/AHA Task Force on Practice Guidelines (Writing Committee to Develop Guidelines for the Management of Patients With Peripheral Arterial Disease) Endorsed by the American Association of Cardiovascular and Pulmonary Rehabilitation; National Heart, Lung, and Blood Institute; Society for Vascular Nursing; TransAtlantic Inter-Society Consensus; and Vascular Disease Foundation J Am Coll Cardiol 2006 47 1239 1312 10.1016/j.jacc.2005.10.009 16545667

[B14] Lin WH Chuang LM Chen CH Yeh JI Hsieh PS Cheng CH An association study of genetic polymorphisms of SLC2A10 gene and type 2 diabetes in Taiwanese population Diabetologia 2006 49 1214 1221 10.1007/s00125-006-0218-3 16586067

[B15] Barrett JC Fry B Maller J Daly MJ Haploview: analysis and visualization of LD and haplotype maps Bioinformatics 2005 21 263 265 10.1093/bioinformatics/bth457 15297300

[B16] Schaid DJ Rowland CM Tines DE Jacobson RM Poland GA Score tests for association between traits and haplotypes when linkage phase is ambiguous Am J Hum Genet 2002 70 425 434 10.1086/338688 11791212 PMC384917

[B17] Mueckler M Family of glucose-transporter genes. Implications for glucose homeostasis and diabetes Diabetes 1990 39 6 11 10.2337/diabetes.39.1.6 2210061

[B18] Cheng CH Kikuchi T Chen YH Sabbagha NG Lee YC Pan HJ Mutations in the SLC2A10 gene cause arterial abnormalities in mice Cardiovasc Res 2008 in press 19028722 10.1093/cvr/cvn319

[B19] Andersen G Rose CS Hamid YH Drivsholm T Borch-Johnsen K Pedersen O Genetic variation of the GLUT10 glucose transporter (SLC2A10) and relationship to type 2 diabetes and intermediary traits Diabetes 2003 52 2445 2448 10.2337/diabetes.52.9.2445 12941788

[B20] Xie X Lu J Kulbokas EJ Golub TR Mootha V Lindblad-Toh K Systematic discovery of regulatory motifs in human promoters and 3' UTRs by comparison of several mammals Nature 2005 434 338 345 10.1038/nature03441 15735639 PMC2923337

[B21] Chaturvedi N Stevens LK Fuller JH Lee ET Lu M Risk factors, ethnic differences and mortality associated with lower-extremity gangrene and amputation in diabetes. The WHO Multinational Study of Vascular Disease in Diabetes Diabetologia 2001 44 Suppl 2 S65 71 10.1007/PL00002941 11587052

[B22] Beks PJ Mackaay AJ de Neeling JN de Vries H Bouter LM Heine RJ Peripheral arterial disease in relation to glycemic level in an elderly Caucasian population: the Hoorn study Diabetologia 1995 38 86 96 10.1007/BF02369357 7744233

[B23] Premalatha G Shanthirani S Deepa R Markovitz J Mohan V Prevalence and risk factors of peripheral vascular disease in a selected south India population Diabetes Care 2000 23 1295 1300 10.2337/diacare.23.9.1295 10977021

[B24] The International HapMap Consortium The International HapMap Project Nature 2003 426 789 796 10.1038/nature02168 14685227

[B25] Yang HC Lin CH Hsu CL Hung SI Wu JY Pen WH Chen YT Fann CS A comparison of major histocompatibility complex SNPs in Han Chinese residing in Taiwan and Caucasians J Biomed Sci 2006 13 489 98 10.1007/s11373-006-9077-7 16544196

