# Supplementary figures and images for: SLC2A10 genetic polymorphism predicts development of peripheral arterial disease in patients with type 2 diabetes. SLC2A10 and PAD in type 2 diabetes
Source: BMC Med Genet. 2010 Aug 25;11:126. doi: 10.1186/1471-2350-11-126 (PMC2939510; doi:10.1186/1471-2350-11-126)

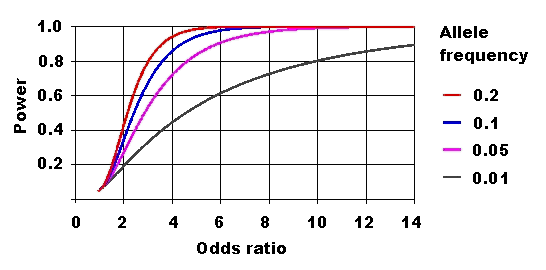

Supplement: Additional file 1 — Supplemental Figure S1. The statistical power of current study to detect a risk allele or haplotype with different odds ratios and frequencies with type I error rate of 0.05. [file 1471-2350-11-126-S1.TIFF]
